# Fungal and Bacterial Communities Exhibit Consistent Responses to Reversal of Soil Acidification and Phosphorus Limitation over Time

**DOI:** 10.3390/microorganisms8010001

**Published:** 2019-12-18

**Authors:** Sarah R. Carrino-Kyker, Kaitlin P. Coyle, Laurel A. Kluber, David J. Burke

**Affiliations:** 1 The Holden Arboretum, Kirtland, OH 44094, USA; laurel.kluber@gmail.com (L.A.K.); dburke@holdenfg.org (D.J.B.); 2 Department of Biology, Case Western Reserve University, Cleveland, OH 44106, USA; kate.coyle@ncsu.edu; 3 Department of Biological Sciences, North Carolina State University, Raleigh, NC 27695, USA

**Keywords:** bacteria, fungi, pH, community structure, 454 sequencing

## Abstract

Chronic acid deposition affects many temperate hardwood forests of the northeastern United States, reduces soil pH and phosphorus (P) availability, and can alter the structure and function of soil microbial communities. The strategies that microorganisms possess for survival in acidic, low P soil come at a carbon (C) cost. Thus, how microbial communities respond to soil acidification in forests may be influenced by plant phenological stage as C allocation belowground varies; however, this remains largely unexplored. In this study, we examined microbial communities in an ecosystem level manipulative experiment where pH and/or P availability were elevated in three separate forests in Northeastern Ohio. Tag-encoded pyrosequencing was used to examine bacterial and fungal community structure at five time points across one year corresponding to plant phenological stages. We found significant effects of pH treatment and time on fungal and bacterial communities in soil. However, we found no interaction between pH treatment and time of sampling for fungal communities and only a weak interaction between pH elevation and time for bacterial communities, suggesting that microbial community responses to soil pH are largely independent of plant phenological stage. In addition, fungal communities were structured largely by site, suggesting that fungi were responding to differences between the forests, such as plant community differences.

## 1. Introduction

Forests are an important terrestrial biome, covering 30% of Earth’s surface and harboring a large percentage of the planet’s plant and animal species [1]. In addition to providing 49% of terrestrial primary productivity [1], forests also provide important ecosystem services and sequester large amounts of carbon (C) in living biomass [2,3], which can offset the effects of anthropogenic emissions on global atmospheric CO_2_ levels and impact global climate change. Despite their importance, forest function is affected by human disturbance, such as landscape fragmentation, overharvesting, and atmospheric air pollution, including acid deposition [4,5,6,7]. Although forest cover has expanded in eastern North America over the last 75 years [8], these forests have developed, and persist, with high levels of acid deposition and the wet and dry deposition of nitrous oxide and sulfur oxide [9]. In fact, there is considerable evidence suggesting that many forests have undergone significant soil acidification since the middle of the 20th century [7,9]. Forest acidification is of particular interest as it can affect the availability of soil phosphorus (P), an essential nutrient that can limit both plant and microbial growth [10]. When soil pH falls below 5, aluminum (Al) is mobilized from soil and can chemically bind to inorganic P (P_i_), making P_i_ generally unavailable for uptake by plants and soil microbes [11].

When soils are acidic, microorganisms elicit a physiological response to cope with the surrounding low pH environment. Such responses include producing membrane proteins and fatty acids for protection from the external environment, producing buffer molecules to maintain internal pH, or through active pumping of hydrogen ions to maintain the proton motive force of the cell membrane [12,13]. In addition to these physiological responses to acidic soil pH, when soils are below a pH of 5, microorganisms are also faced with low soil P_i_ due to mobilized Al (as described above). Soil microbes can compensate for the limited availability of P_i_ by acquiring more P from organic sources (P_o_) through the production of phosphatases or by producing organic acids that liberate P_i_ from metal complexes [14,15,16]. However, the production of cellular molecules necessary for survival under acidic soil pH and low P availability come at an energetic and C cost to the microbes (e.g., see [17]). Soil pH and P availability are known to have large impacts on soil microbial communities, including community structure (e.g., [18,19,20,21,22,23]). Indeed, the large body of literature correlating soil pH with soil fungal and bacterial community structure, diversity, and biomass (e.g., [21,23,24,25]) suggests that soil pH may be the primary factor influencing community changes in a variety of ecosystems. Besides community structure changes, reductions in microbial biomass or respiration have been found in soils with acidic pH [26] or limited P availability [27,28]. It is plausible, and we have previously suggested [26], that these changes to microbial community structure and microbial biomass reflect an energetic and C cost associated with survival in low pH and low P soils.

The flow of photosynthetic C from temperate forest plants to soil microorganisms changes over the course of a calendar year as plant phenological stages change. During the growing season, plants actively transfer C to roots and rhizosphere soil in the form of relatively labile root extracts, while during the dormant season when plants are not actively fixing C, microbial communities become more dependent on recalcitrant forms of C present in litter and organic matter [29,30]. Such changes in labile C input from plants can have large effects on soil microbial activities [31]. As described above, we have previously found community changes when soil pH and soil P were experimentally elevated for mycorrhizal and root-associated fungi on mature tree roots [20,26], but these investigations were conducted only during the growing season in late summer. It is plausible that the availability of soil C over time (i.e., growing season compared to dormant season) may interact with the response (i.e., community structure change) of soil microorganisms to soil acidification and P limitation. However, the response of microbial communities to soil pH and P availability in conjunction with changes to plant phenological stage has been largely unexplored.

Here we describe a study that examined the microbial community response to soil acidification and P limitation over one calendar year within an ecosystem level manipulative experiment in northeastern Ohio. Within each plot, the soil was amended such that it had elevated pH, elevated P, elevated pH + P, or was an untreated control [20,26,32]. We used 454 sequencing to examine the response of soil fungal and bacterial communities to altered soil chemistry over five sampling times corresponding to annual plant phenological stages. We hypothesized that: (1) bacterial and fungal communities would be affected by changes to soil P_i_ during the growing season since P_i_ is expected to limit microbial activity and growth while labile C availability is high; and (2) since fungi are the primary decomposers in temperate forests [33], fungal communities would respond strongly to P_i_ limitation during the dormant season when pH and P_i_ could create a dual limitation (P and C) during a time of high decomposition activity.

## 2. Materials and Methods

### 2.1. Site Description

Our experiment consisted of 36 experimental plots (measuring 20 m × 40 m; 800 m^2^) that were located in three mature temperate deciduous forests within Northeastern Ohio, USA. The 36 plots were located in two forest stands at The Holden Arboretum (Kirtland, Ohio) and one forest stand at Squire Valleevue and Valley Ridge Farms (Case Western Reserve University, Hunting Valley, Ohio), with 12 plots per forest. Forests are dominated by oak (*Quercus* spp.), maple (*Acer* spp.), and American beech (*Fagus grandifolia*) trees. This region has an average temperature of 8.1 °C, an average of 120 cm of precipitation and soils are loamy to silty loams with an average soil pH of 4.3 for glaciated forests. Additional description of the plots can be found in DeForest et al. [32].

### 2.2. Experimental Design

Beginning in the autumn of 2009, treated plots received either a direct application of crushed limestone (Hi-Ca lime; high calcium to magnesium ratio) to increase soil pH or triple super phosphate (TSP) to elevate soil P_i_ availability, or both in combination for an elevated pH + P treatment. The elevated pH and elevated pH + P plots initially received 11.4 mg ha^−1^ of Hi-Ca lime (The Andersons, Maumee, OH, USA) in an effort to raise soil pH to within a range of 5.8 to 6.2 for the top 5 cm. This target pH was chosen because exchangeable aluminum is immobilized within this soil pH range. Additional lime applications were made in the fall of 2010 to maintain the target soil pH. To increase P availability, the elevated P and elevated pH + P plots received approximately 42 kg P ha^−1^ in 2010 prior to our sampling and an additional 34 kg P ha^−1^ in spring 2011 (following our spring sampling, but prior to our early summer sampling; see below) as TSP (The Andersons, Maumee, OH, USA). Treatments effectively raised soil pH by approximately 1.5 units and available P_i_ (i.e., resin P) by approximately 6-fold (see [20,26] for detailed soil chemistry data collected at the start and end of the sampling described here, respectively).

### 2.3. Soil Sampling

**Figure 1:**
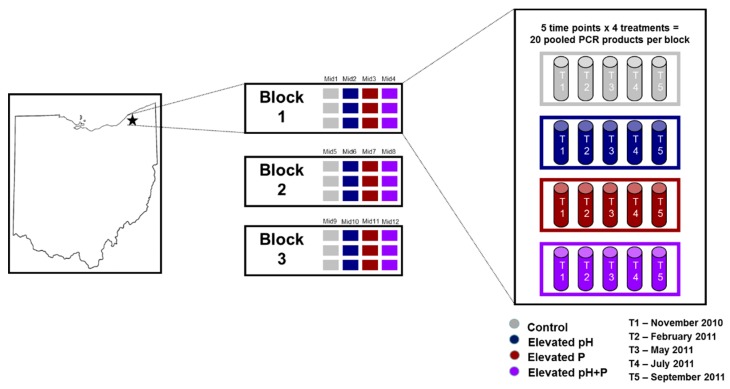
Diagram of the study design, which indicates the three forest blocks. Within each forest block were twelve plots (three of each treatment; shown in different colors) for a total of 36 plots. Soil from each plot was sampled five times throughout a calendar year (indicated as T1–T5) for a total of 180 soil samples that were used for DNA extraction. The DNA was then pooled within each treatment of each block for 20 DNA samples per block (a schematic of the DNA samples for one block is shown) and 60 DNA samples total that were sequenced. MID = multiplexing identifier.

Soils were collected from each of the 36 plots at five time points within a calendar year for a total of 180 samples (Figure 1). The collections were made in November 2010 (in fall; after trees dropped their leaves), February 2011 (winter), May 2011 (spring; after tree leaf out), July 2011 (early summer), and September 2011 (late summer). These time points were chosen because they corresponded with distinct stages of tree phenology, including leaf out and litter fall, and should reflect changes in microbial resource availability (i.e., C) over the year. At each time point, 15 soil cores (2.5 cm diameter, 5 cm depth) were randomly sampled within each plot, pooled, sieved to 2 mm, and stored at −70 °C until used for DNA extraction.

### 2.4. DNA Extraction

DNA was extracted from approximately 0.5 g of soil for each of the 180 samples using a standard phenol/chloroform extraction procedure described in Burke et al. [18]. DNA was quantified using a NanoDrop spectrophotometer (Thermo Fisher Scientific, Waltham, MA, USA) and, for each time point, the three treatment replicates per forest were pooled on an equimolar basis for a final template concentration of 25 ng/μL. This resulted in a total of 60 DNA samples (12 for each time point; Figure 1).

### 2.5. PCR

The fungal community was analyzed by PCR amplification of the internal transcribed spacer (ITS)1 region of the rRNA gene using the forward primer ITS1-F (CTTGGTCATTTAGAGGAAGTAA) [34] and the reverse primer ITS2 (GCTGCGTTCTTCATCGATGC) [35], which have been proven effective in previous pyrosequencing studies of fungal diversity in forest soils [36]. Attached to the ITS1-F primer were multiplexing identifiers (MIDs) to allow for separation of sequence reads into treatment × forest combinations. A total of 12 MIDs were used and were consistently assigned to a treatment/forest DNA extract across time points. Each primer also had a Genome Sequencer (GS) FLX Titanium Primer attached to allow for sequencing with primer A attached to the forward and primer B attached to the reverse. For the fungal PCRs, each 25 μL reaction contained 22.5 μL Platinum^®^ PCR SuperMix High Fidelity (Thermo Fisher Scientific), 1 μL template, 0.4 μg/μL bovine serum albumin, and 0.1 μM of each primer. Thermocycling conditions were 3 min at 94 °C, followed by 33 cycles of 94 °C for 45 s, 50 °C for 30 s, and 72 °C for 60 s, with a final extension of 72 °C for 10 min. For bacterial community structure analysis, the 16S gene was PCR-amplified with the primer set 27F (AGAGTTTGATCCTGGCTCAG) and 338R (TGCTGCCTCCCGTAGGAGT) [37] with the GLS FLX Titanium Primer A and Primer B, respectively, attached to allow for sequencing and MIDs also attached to the 338R primer to allow for separation into treatment × forest combinations as described above. The 25 μL bacterial PCR reactions contained 17.5 μL Platinum^®^ PCR SuperMix High Fidelity, 1 μL template, 0.5 μg/μL bovine serum albumin, and 0.1 μM of each primer and thermocycling conditions were 3 min at 94 °C, followed by 30 cycles of 94 °C for 45 s, 50 °C for 30 s, and 72 °C for 60 s, with a final extension of 72 °C for 10 min. This resulted in 120 fungal PCR products and 120 bacterial PCR products. Bacterial and fungal PCR products were combined at a 3:1 bacterial:fungal ratio to account for greater bacterial abundance while allowing all PCR products to be run on the same 454 sequencing plate.

### 2.6. 454 Sequencing

Sequencing was completed on the Genome Sequencer FLX 454 System through Case Western Reserve University’s Genomics Core with each of the five time points run on a separate 1/8 section of the plate. This allowed for separation between the time points, while the 12 MIDs used during PCR allowed for separation of the treatment × forest combinations.

### 2.7. Sequence Analysis

Sequences were demultiplexed using Galaxy with usegalaxy.org [38,39,40] where primer sequences were used to separate fungal and bacterial sequences and MIDs were used to separate the different treatment × forest samples. Following demultiplexing, fungal and bacterial sequence reads were analyzed within the UPARSE pipeline [41]. The USEARCH software [42] was used to trim sequences to a fixed base pair length to allow for global alignment; fungal sequences were trimmed to 200 bp and bacterial sequences were trimmed to 250 bp. Sequences were filtered for quality with the maximum expected error parameter (fastq_maxee) set to 3. The “sortbysize” command was used to remove singletons (i.e., sequences that occurred only once) and the “cluster_otus” command was used for operational taxonomic unit (OTU) clustering at 97% similarity with the UCLUST algorithm [42]. Chimeras were removed with reference-based chimera filtering using the UCHIME algorithm [43] and screening against the UNITE database [44,45] for fungi and the Genomes OnLine Database (GOLD) database [46] for bacteria. Taxonomy was assigned within the assign_taxonomy.py command of the Quantitative Insights Into Microbial Ecology (QIIME) pipeline [47] with Basic Local Alignment Search Tool (BLAST) comparisons [48] to the UNITE database [44,45] for fungi and UCLUST comparisons [42] to the GreenGenes database [49,50] for bacteria. Finally, OTUs containing only one sequence read (i.e., singletons) were removed from the dataset, as such OTUs can represent sequencing errors [51]. Sequences are publicly available through the Sequence Read Archive at the National Center for Biotechnology Information (NCBI) site under BioProject number PRJNA377728 and BioSample numbers SAMN06471527 through SAMN06471531.

### 2.8. Statistical Analysis

Matrices of fungal and bacterial OTUs were further processed by removing OTUs that were found in only one sample and OTUs that did not have meaningful taxonomic matches (i.e., the pipeline returned the taxonomic identity as “unassigned” or “no blast hit” and no domain match, as well as the bacterial OTU that matched with “chloroplast”). While this can potentially remove rare taxa, it also removed OTUs that are potential sequencing errors and non-target OTUs that did not match at a domain level with Bacteria or at a kingdom level with Fungi. A total of 13,592 fungal sequence reads and 80,854 bacterial sequence reads representing 573 fungal and 1967 bacterial OTUs met our quality control parameters and were used for statistical analysis. To compare fungal and bacterial communities among treatments and across time points, nonparametric permutation procedure (PERMANOVA) and principle components analysis (PCoA) were conducted using the *adonis* and *betadisper* commands, respectively, in the vegan package (v2.5.5) [52] of R (v3.5.3) [53]. Both PERMANOVA and PCoA analyses used the Bray-Curtis distance measure. Sequence counts were normalized with the trimmed mean of M (TMM) method of the edgeR package (v3.18.1) [54,55] prior to statistical analyses, which has shown to be a useful method for comparing sequencing libraries of varying size [56]. In order to visualize changes in taxa between treatments and time points, heat maps were made with the Heatplus package (v2.28.0) [57], also in R. Dendrograms associated with the heat maps used the Bray-Curtis distance measure (calculated in the vegan package) to cluster samples and included OTUs that had at least one instance of 1% relative abundance for bacterial taxa and 2% relative abundance for fungal taxa. To further analyze fungal communities, fungal OTUs were also parsed into functional guilds based on their taxonomic assignment using FUNGuild (v1.0) [58]. Only guild assignments that had confidence rankings of “highly probable” and “probable” were included in statistical analysis and the dataset was further subset into guilds that included “Plant Pathogen”, “Ectomycorrhizal”, “Saprotroph”, and “Ericoid mycorrhizal” according to FUNGuild. Linear mixed effect models were used to test for differences in the relative sequence abundance of bacterial taxa and functional guilds between treatments and sampling time points using the lme command in the R package nlme (v3.1.137) [59], where forest block was used as the random effect. To test for differences between the individual treatments (control, elevated pH, elevated P, and elevated pH + P) and time points, multiple comparisons were calculated with the *glht* command in the multcomp package of R (v1.4.10) [60]. Graphs were made using ggplot2 (v3.2.1) [61] in R.

## 3. Results

### 3.1. Bacterial Community Structure

**Figure 2:**
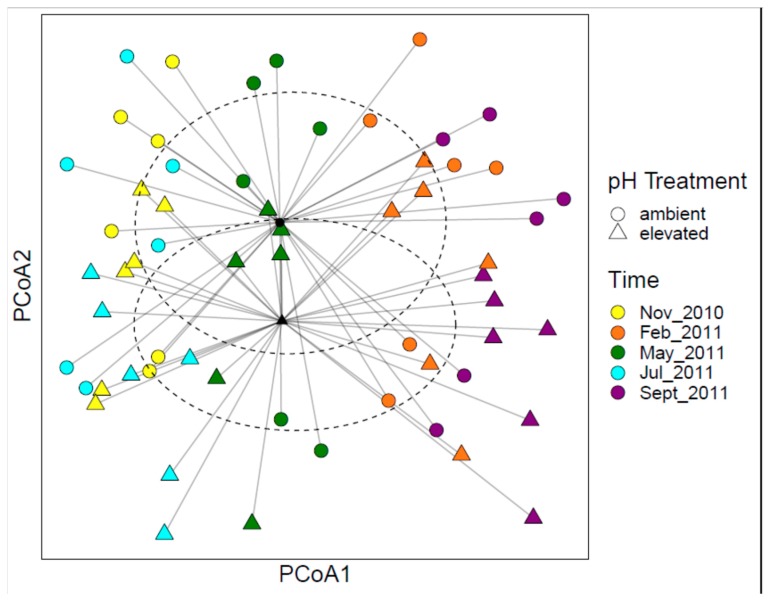
Principle component analysis (PCoA) ordination of the bacterial community structure changes between pH treatments and over time. Different colors represent different time points, while the shapes represent different pH treatments. Centroids and ellipses for the pH treatments are also shown.

**Figure 3:**
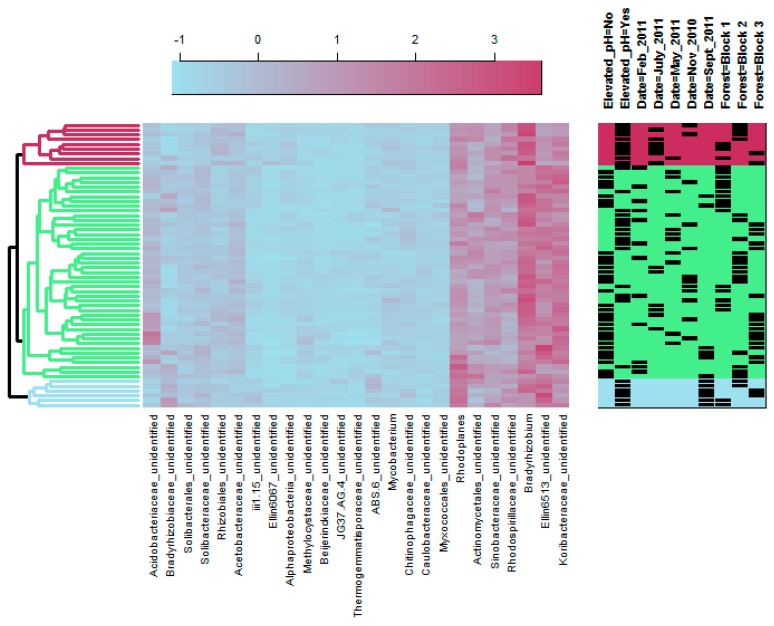
Heatmap of the bacterial taxa present at 2% relative abundance or above. Taxa were identified to genus if possible and then the lowest taxonomic rank available. Three clusters of samples were found based on bacterial taxa and are shown in three colors. Treatments, time points, and forest blocks are indicated by the shaded rectangles on the right. The scale of the color legend is log base 10 of the numbers shown.

PERMANOVA results indicated that bacterial communities were significantly affected by pH treatment and time of sampling (Table 1). PCoA showed clear shifts in bacterial community structure over time, as well as between elevated and ambient soil pH conditions (Figure 2). The bacterial taxa that shifted with pH elevation were dependent on the time of sampling, as shown by the dendrogram on the bacterial heat map (Figure 3). Two clusters of elevated pH samples were apparent; the top cluster (shown in maroon) included samples collected largely in July, but also in May and November, while the bottom cluster (shown in light blue) included samples collected only in September (Figure 3). In general, elevated pH plots in September had higher relative sequence numbers for the genus *Rhodoplanes*, while elevated pH plots in July and November had higher relative sequence numbers for the genus *Bradyrhizobium* compared to ambient pH plots (Figure 3). Sequences matching the order Ellin6519 (phylum *Acidobacteria*) were relatively higher in elevated pH plots in September, but relatively lower in elevated pH plots in July compared to other time points (Figure 3). OTUs that matched with the family *Koribacteraceae* were generally in lower relative abundance for both elevated pH dendrogam clusters compared to the cluster with all ambient pH samples (shown in green; Figure 3). OTUs that matched with the family *Acidobacteriaceae* were generally in higher relative abundance for the middle cluster, which contained all ambient pH plots.

### 3.2. Fungal Community Structure

**Figure 4:**
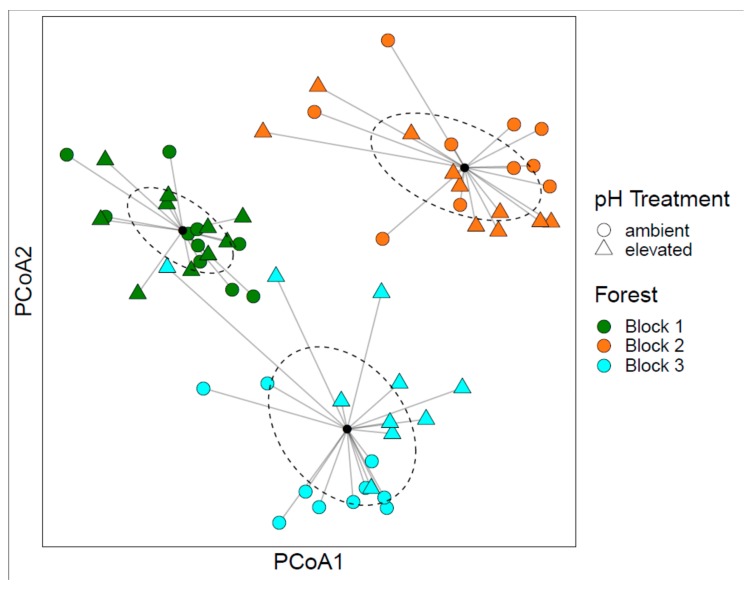
PCoA ordination of the fungal community structure changes between pH treatments and forest blocks. Different colors represent different forest blocks, while the shapes represent different pH treatments. Centroids and ellipses for the blocks are also shown.

**Figure 5:**
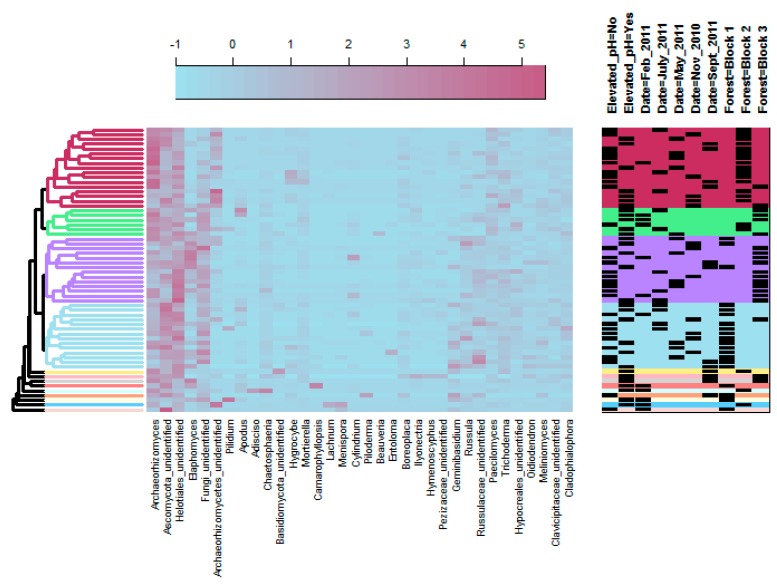
Heatmap of the fungal taxa present at 5% relative abundance or above. Taxa were identified to genus if possible and then the lowest taxonomic rank available. Four main clusters of samples were found based on bacterial taxa and are shown in three colors. Treatments, time points, and forest blocks are indicated by the shaded rectangles on the right. The scale of the color legend is log base 10 of the numbers shown.

**Figure 6:**
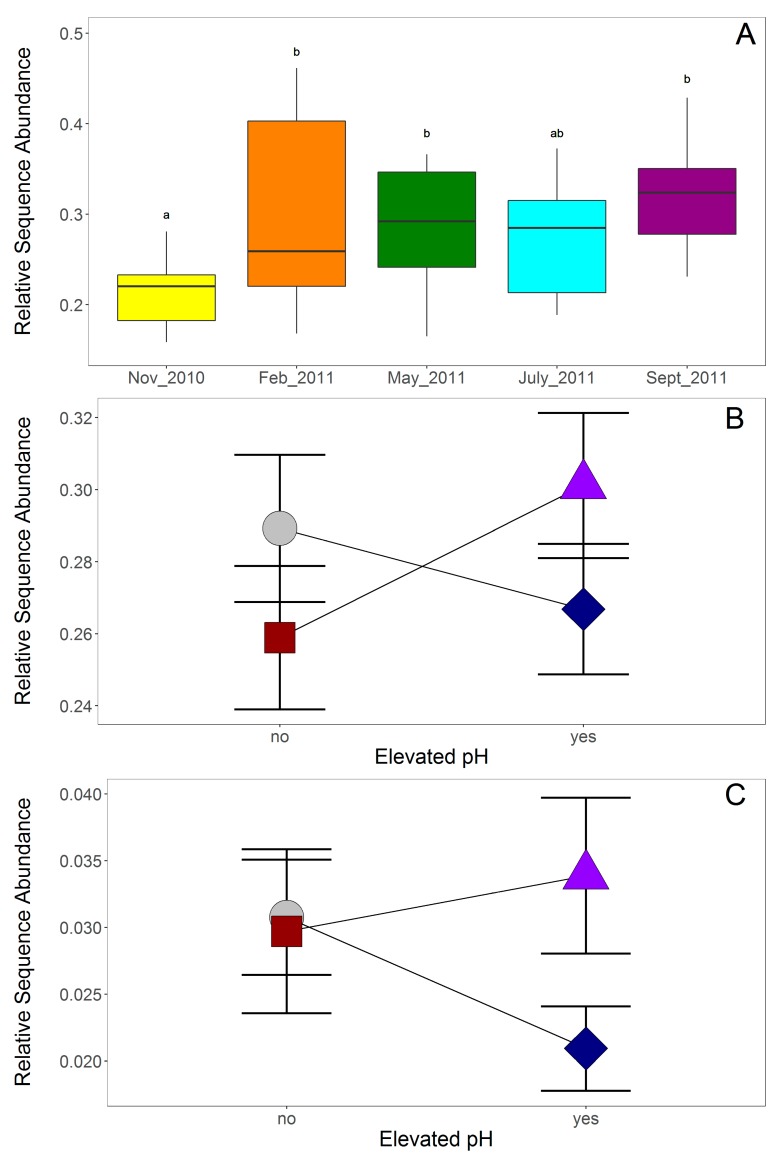
Relative sequence numbers for saprotrophic fungi and represented as (**A**) box and whisker plots at each collection time point or (**B**) mean ± standard error for each treatment and (**C**) ericoid mycorrhizal fungi represented as mean ± standard error for each treatment. For plot A, different letters denote time points that differed significantly. For plots B and C, treatments are represented by gray circles for Control, blue diamonds for Elevated pH, red squares for Elevated P, and purple triangles for Elevated pH + P (colors are as in Figure 1). Elevated and ambient pH plots are separated along the x-axis and lines connect elevated P plots and ambient P plots to each other to show the significant interaction. No letters are shown, as the treatments did not differ from one another significantly.

Similar to the bacterial community results, PERMANOVA on the fungal community structure indicated that fungal communities were significantly affected by pH treatment and time of sampling (Table 2). However, PCoA showed samples clustering in ordination space mostly by forest block, suggesting that fungal community changes were largely driven by the location of sampling (Figure 4). Separation between elevated pH (triangles) and ambient pH (circles) were generally apparent for each forest block (Figure 4), which confirms the significant effect of pH treatment on fungal community structure with PERMANOVA. Because of the strong effect of location on fungal communities, taxonomic differences between soil treatments or over time were more nuanced (Figure 5). However, when fungal taxa were grouped by functional guild, differences in the relative abundance of saprotrophic fungi were apparent over time (Table 3, Figure 6A). In addition, significant interactions between pH and P elevation were found for the relative abundance of saprotrophic fungi and ericoid mycorrhizal fungi (Table 3), however, multiple comparisons showed no significant differences between the individual treatments (Figure 6B,C). The forest blocks differed in their relative abundance of saprotrophic fungi, ectomycorrhizal (ECM) fungi, and ericoid mycorrhizal (ERM) fungi (Figure S1). These differences in functional guilds may be driven by the dominant tree species, since the forest block with the lowest abundance of ectomycorrhizal fungal sequences is also the forest block with the highest number *Acer* trees (sugar maple and red maple), which form associations with arbuscular mycorrhizal fungi and could explain the lower abundance of the ectomycorrhizal and ericoid mycorrhizal functional guilds.

## 4. Discussion

Contrary to our predictions, this study found little evidence to support our hypothesis that the microbial community response to soil pH and P_i_ limitation would be altered by changes in plant phenological stage and the potential availability of labile C. Bacterial and fungal communities both demonstrated temporal variation, however the response of these communities to soil pH and P_i_ availability were generally consistent over time, especially for soil fungi and with the exception of two bacterial genera in the Alphaproteobacteria family. Soil pH treatment altered both bacterial and fungal communities, while soil P_i_ availability had no effect on either community.

The effects of pH on microbial community structure, diversity and richness has been established in a number of previous studies (e.g., [23,25]), although the cause of microbial response to pH cannot be easily determined. Because pH can have direct and indirect effects on soil microbes, their response to pH could reflect both a response to biochemical conditions in the surrounding soil [62] as well as biogeochemical processes that affect nutrient availability [63]. For example, changes in pH can affect nutrient and chemical availability, especially for P and Al which can interact to affect overall P nutrient availability [64]. The majority of microbes, though, have a pH range of only 2–3 units wide, above or below which they cannot survive [62,65] and need to maintain cytoplasmic pH near an optimum for cell survival and activity [12]. It is, therefore, unsurprising that a change in pH of 2 units in the current study caused significant shifts in both fungal and bacterial communities.

### 4.1. Fungal Community Response to pH Treatment

In previous work, we found that elevating pH altered root-associated fungal communities and increased mycorrhizal colonization and fungal biomass in soil during the growing season [20,26]. Those results, along with the results of the current study, are in agreement with a large body of literature suggesting that pH has substantial effects on soil fungi [18,23,66,67,68,69]. In the present study, we found that changes to fungal communities with elevated pH are not restricted to the time of active plant growth but rather persist throughout the year. Although some authors have found evidence for increased turnover of mycorrhizal fungi under acidified conditions [70], our study found that temporal changes in fungal communities were similar in both ambient and elevated pH plots. This can be visualized by the fungal heat map (Figure 5), which shows the clusters comprised of samples from both pH treatments and all time points. Further, in the current study, the relative abundance of ECM fungal sequences did not differ significantly over time (Table 3), but saprotrophic fungal sequences did (Figure 6A). Abundance and biomass of saprotrophic fungi are known to vary as litter quality changes [71] and with invertebrate grazing [72]. As such, the temporal changes observed in the current study are to be expected for saprotrophic fungi. However, our study is contrary to many other studies which show significant temporal variation and high turnover for mycorrhizal fungal taxa [73]. The current study, though, examined bulk soil and not live root tips, which could explain this discrepancy. The soil samples we collected had between 1.9% and 36.5% ectomycorrhizal fungal sequences (an average of 13.6%), while saprotrophic fungi were more abundant and comprised between 15.8% and 46.1% (an average of 27.9%) of DNA sequences.

Fungal communities in the current study were structured more by the forest block than by the treatments or time. This is evident in the heatmap (Figure 5), which shows higher relative abundance of the mycorrhizal genus *Elaphomyces* in forest block 3 and of the non-mycorrhizal genus *Archaerhizomyces* in forest block 2 compared to the other forest blocks. At forest block 2, *Archaerhizomyces* was typically found in plots at ambient pH. We previously found that *Archaerhizomyces* was a significant indicator of ambient pH plots (including the same ones in forest block 2) as part of a larger study [26]. In a recent study by Pinto-Figueroa [74], *Archaerhizomyces* relative abundance was influenced by a number of climate and edaphic properties. In the current study, the climatic conditions were consistent among our forest blocks, as they were all located at a similar latitude (see glaciated sites of Table 1 in DeForest et al. [32]) and it is difficult to discern if edaphic differences between the forest blocks were influencing the relative abundance of *Archaerhizomyces* since similar soil characteristics were not measured. However, it is plausible that soil pH may interact with other soil properties to influence the distribution of this largely uncultured but widespread fungal genus and future studies could help to disentangle these. We have previously documented that the forest blocks differ in their basal area of tree genera (see glaciated sites of Table 1 in DeForest et al. [32]), with *Acer* spp. representing over 68% of the total basal area in forest block 2 compared to around 30% in the other forest blocks and *Fagus* spp. (primarily *F. grandifolia*) representing over 21% of the total basal area in forest block 3, compared to less than 6% in the other forest blocks. These changes in tree dominance could be influencing the fungal taxa at each site. For example, the low relative abundance of ectomycorrhizal fungal sequences in forest block 2 (Figure S1) could be related to the dominance of *Acer* trees, as these trees form relationships with arbuscular mycorrhizal fungi, while ectomycorrhizal-associated tree species, such as those in the *Quercus* and *Fagus* genera, were in low abundance (16.1% and 5.6% of total basal area, respectively; see DeForest et al. [32]). Plant host [75,76,77] and microsite variability [78] are important drivers of fungal community structure and diversity, especially for plant-associated fungi. Our data suggest that forest block was the most important factor influencing fungal community composition, which is similar to other multi-year studies on fungal community structure [79]. Thus, the current study builds on the literature showing that fungal communities in forests are structured largely by the availability of suitable plant hosts and high spatial heterogeneity at small scales, such as the stand level (reviewed by Baldrian [80]).

### 4.2. Bacterial Community Response to pH Treatment

Similar to our results with fungal communities, bacterial communities were also affected by elevating pH, which is consistent with many other studies that have observed an effect of soil pH on bacterial community structure (e.g., [21,23,25,81]). In the current study, the taxa that responded to elevated soil pH are generally in agreement with Lauber et al. [21] where the relative abundance of Acidobacteria was higher at low pH conditions (consistent with *Acidobacteriaceae* and *Koribacteraceae* OTUs of the heat map; Figure 3). It should be noted that although Rousk et al. [23] found that some Acidobacteria subgroups were more abundant at low pH, others were more abundant at elevated pH, including subgroup 2. This could explain the higher relative abundance at elevated soil pH we found of the OTU matching with the Acidobacteria order Ellin6513, which is an undescribed taxa in subdivision 2 seen during the September sampling (see Figure 3). Although Lauber et al. [21] did not show clear effects of pH on Alphaproteobacteria, we found higher relative abundance of the genera *Rhodoplanes* and *Bradyrhizobium* in elevated pH plots at different time points (see Figure 3). This is similar to Rousk et al. [23] who found an increase in relative abundance of proteobacterial groups with increasing soil pH and Bartram et al. [82] who found a number of bacterial genera within the Alphaproteobacteria associated with neutral soil pH, including *Rhodoplanes* and another rhizobial genus (although from a separate family), *Mesorhizobium*. It has been shown that Alphaproteobacteria are associated with C availability [83], which could explain the temporal variation associated with pH treatment for *Rhodoplanes* and *Bradyrhizobium*, which were relatively higher in September and July/November, respectively. Thus, these genera increased in relative abundance primarily during the growing season when plants are providing photosynthates for their mutualists, like nitrogen-fixing rhizobia.

### 4.3. Microbial Community Response to P Treatment

The plots of the current study were also manipulated to elevated soil P_i_ availability and the effects of this elevation on microbial communities in forests are still not well understood. We found that fungal community response to P addition was not strong, with no effect of P addition on either fungal or bacterial community structure. Experimental addition of P in plots at Hubbard Brook Experimental Forest found few effects of P addition on fungal biomass but overall microbial community composition did change in P addition plots relative to control plots [84]. Forests in southern China showed variable responses to P addition, with fungal biomass increasing and microbial community structure changing in response to P addition in old-growth evergreen forest plots, whereas microbial communities and biomass in pine and mixed broadleaf forests showed no response to P addition [85]. In a meta-analysis of field studies, Treseder [86] found some support for P effects on mycorrhizal fungal communities, but the effects varied among studies. It should be noted that only two studies examining ectomycorrhizal fungal communities and P addition experiments were included in Treseder [86], highlighting the general lack of studies exploring P effects on ectomycorrhizal fungi of forests. In our previous work, we found significant correlations between available forms of soil P, specifically P_o_, and ectomycorrhizal community structure in an old growth beech maple forest, but this correlation was seasonally dependent and evident only during late summer sampling [18]. We suggest that the weak effect of TSP application on fungal communities in the current study could be tied to microsite variation in resource availability. For example, in an old growth forest where fungal communities were correlated with soil P, it was site location that was the largest factor explaining variation in these fungal communities, suggesting that microsite variation in soil P was related to fungal community structure [78]. The current study did show significant interactions for soil pH and P treatments for the relative abundance of saprotrophic and ERM fungi (a group known to degrade organic material [87]; Figure 6B,C), suggesting that alleviating both soil acidity and P limitation together could increase the abundance of these functional groups. However, multiple comparisons showed no significant differences between any of the individual treatments, indicating that the effects of P_i_ manipulation overall on fungi were weak. Bacterial communities in the current study responded similarly to fungal communities to P addition with no changes in community structure. The consistent response of bacteria and fungi to changes in soil P is a result not unlike that of Liu et al. [85], in that both fungal and bacterial biomass responded similarly to P addition in old growth evergreen forests. Groffman and Fisk [84] saw no effect of P addition on microbial biomass in a temperate hardwood forest, although P addition did alter the overall microbial community structure compared to control plots.

### 4.4. Summary

In summary, we found that both bacterial and fungal communities responded to changes in soil pH within our temperate forests and that these changes were generally not influenced by the season of sampling. One exception to this was the relative abundance two genera of Acidobacteria, which increased at higher pH primarily during the growing season. Contrary to our hypotheses, we found no effect of changing P_i_ availability on microbial community structure. However, we did find a strong influence of site on fungal communities. Our data are consistent with a number of previous investigations showing soil pH to have a strong effect on structuring microbial communities and that the heterogeneity inherent of many forests is a potentially strong driver of soil fungal community composition in particular.

## Figures and Tables

**Table 1:** Three-way nonparametric permutation procedure (PERMANOVA) showing the effects of pH treatment, phosphorus (P) treatment, and time of sampling, along with the interactions, on soil bacterial community structure. The samples were blocked by forest using the strata option.

	df^†^	SS^†^	Mean Sqs^†^	*F*-Value	R^2^	*p*-Value *
pH	1	0.51	0.51	3.99	0.048	**<0.001**
P	1	0.13	0.13	0.98	0.012	0.26
Time	4	3.46	0.87	6.78	0.33	**<0.001**
pH:P	1	0.15	0.15	1.17	0.014	0.14
pH:Time	4	0.56	0.14	1.10	0.053	*0.098*
P:Time	4	0.33	0.083	0.65	0.032	0.97
pH:P:Time	4	0.34	0.085	0.67	0.032	0.96
Residuals	80	5.11	0.13		0.48	
Total	119	10.60			1	

^†^df: degrees of freedom; SS: sequential sums of squares; Mean Sqs: mean squares. * Significant *p*-values at α = 0.05 are in bold; *p*-values approaching significance at α = 0.1 are in italics.

**Table 2:** Three-way nonparametric permutation procedure (PERMANOVA) showing the effects of pH treatment, P treatment, and time of sampling, along with their interactions, on soil fungal community structure. The samples were blocked by forest using the strata option.

	df^†^	SS^†^	Mean Sqs^†^	*F*-Value	R^2^	*p*-Value *
pH	1	0.49	0.49	1.53	0.025	**<0.001**
P	1	0.28	0.28	0.87	0.014	0.27
Time	4	3.32	0.83	2.59	0.17	**<0.001**
pH:P	1	0.24	0.24	0.76	0.012	0.67
pH:Time	4	0.94	0.23	0.73	0.047	0.94
P:Time	4	0.86	0.21	0.67	0.043	1.00
pH:P:Time	4	0.82	0.20	0.64	0.041	0.99
Residuals	40	12.82	0.32		0.65	
Total	59	19.77			1	

^†^df: degrees of freedom; SS: sequential sums of squares; Mean Sqs: mean squares. * Significant *p*-values at α = 0.05 are in bold.

**Table 3:** Linear mixed-effects models showing the effects of pH treatment, P treatment, and time of sampling, along with their interactions, on the relative sequence abundance of different fungal guilds. The forest blocks were the random effect in the models.

	numDF^†^	denDF^†^	*F*-Value	*p*-Value *
**Saprotroph**				
pH	1	38	0.47	0.50
P	1	38	0.019	0.89
Time	4	38	6.57	**<0.01**
pH:P	1	38	5.02	**0.031**
pH:Time	4	38	2.56	0.054
P:Time	4	38	0.096	0.98
pH:P:Time	4	38	1.38	0.26
**Ericoid Mycorrhizal Fungi**				
pH	1	37	0.012	0.91
P	1	37	0.70	0.41
Time	4	37	0.75	0.56
pH:P	1	37	4.71	**0.036**
pH:Time	4	37	0.32	0.86
P:Time	4	37	0.26	0.90
pH:P:Time	4	37	0.90	0.47
**Ectomycorrhizal Fungi**				
pH	1	38	0.23	0.64
P	1	38	0.80	0.38
Time	4	38	1.46	0.23
pH:P	1	38	0.026	0.87
pH:Time	4	38	0.15	0.96
P:Time	4	38	1.41	0.25
pH:P:Time	4	38	0.67	0.62
**Pathogen**				
pH	1	36	0.0013	0.97
P	1	36	0.85	0.36
Time	4	36	1.49	0.23
pH:P	1	36	2.24	0.14
pH:Time	4	36	0.41	0.80
P:Time	4	36	2.21	0.087
pH:P:Time	4	36	0.77	0.55

^†^numDF: numerator degrees of freedom; denDF: denominator degrees of freedom. * Significant *p*-values at α = 0.05 are in bold.

## Supplementary Materials

The following are available online at https://www.mdpi.com/2076-2607/8/1/1/s1, Figure S1: Relative sequence numbers of saprotrophic, ectomycorrhizal, and ericoid mycorrhizal fungi in each forest block.
