# Supplementary material for: Fungal and Bacterial Communities Exhibit Consistent Responses to Reversal of Soil Acidification and Phosphorus Limitation over Time
Source: Microorganisms. 2019 Dec 18;8(1):1. doi: 10.3390/microorganisms8010001 (PMC7022789; doi:10.3390/microorganisms8010001)
Supplement: Supplementary file 1 [file microorganisms-08-00001-s001.pdf]

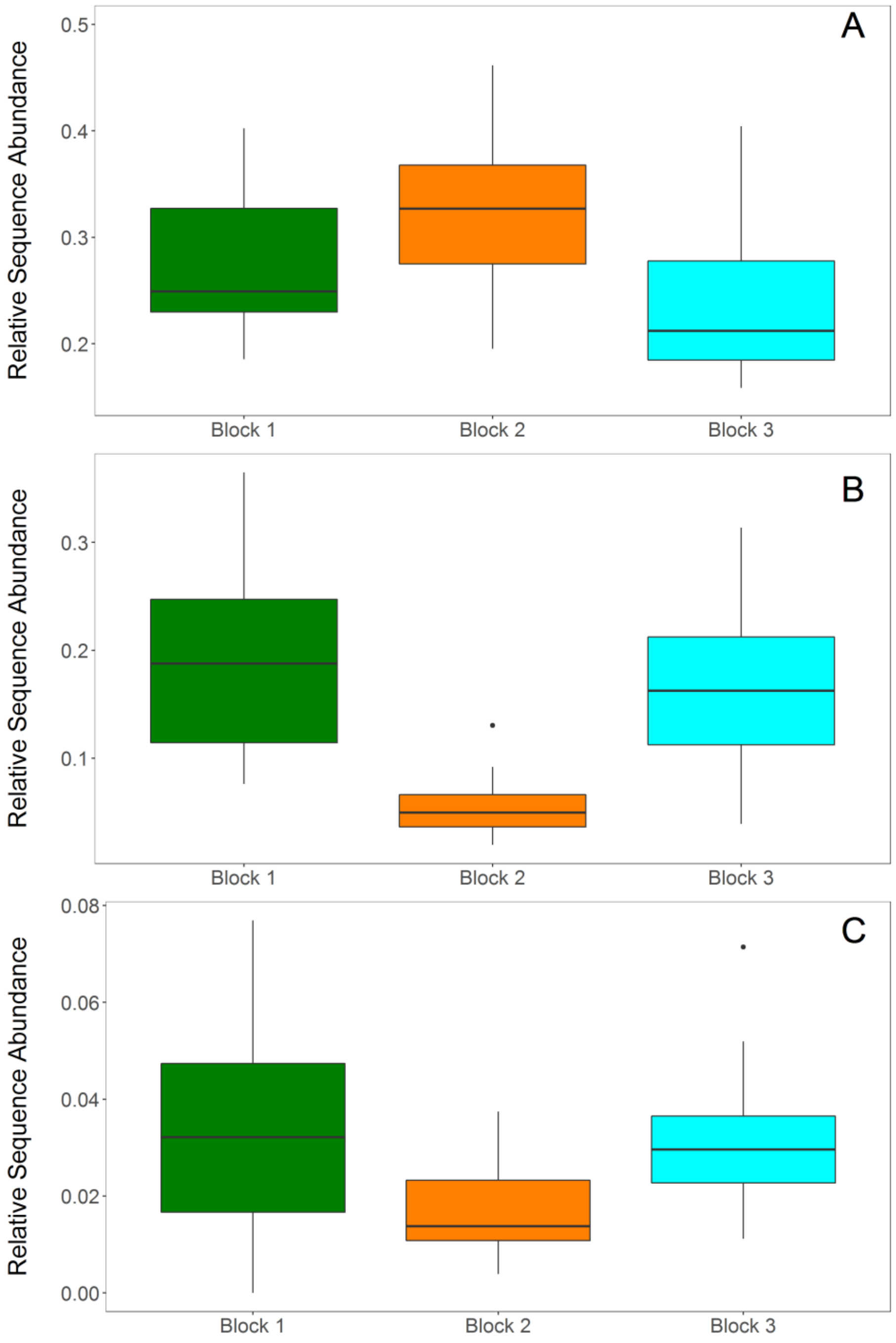


**Figure S1.** Relative sequence numbers of A) saprotrophic, B) ectomycorrhizal, and C) ericoid mycorrhizal fungi at each block. Block 2 had lower relative numbers of mycorrhizal taxa and higher relative numbers of saprotrophic taxa.
